# Predictive Factors of Suicidal Ideation in Spanish University Students: A Health, Preventive, Social, and Cultural Approach

**DOI:** 10.3390/jcm12031207

**Published:** 2023-02-02

**Authors:** Cristina Lázaro-Pérez, Pilar Munuera Gómez, José Ángel Martínez-López, José Gómez-Galán

**Affiliations:** 1 Faculty of Social and Health Sciences, University of Murcia, 30100 Murcia, Spain; 2 Faculty of Social Work, Complutense University of Madrid, 28223 Madrid, Spain; 3 Faculty of Social Work, University of Murcia, 30100 Murcia, Spain; 4 Faculty of Education and Psychology, University of Extremadura, 06006 Badajoz, Spain

**Keywords:** suicidal ideation, university students, university, suicide, young people, predictive factors, prevention

## Abstract

Suicide, as the ultimate expression of suicidal ideation, has accompanied human beings throughout history within specific social and cultural contexts. However, in recent decades the increase in suicides, especially in developed countries after the Second Demographic Transition and the rise of postmaterialist values, has been increasing in the youth population. This study is created from a quantitative perspective and aims to determine the predictors of suicidal ideation in university students in Spain. The fieldwork was carried out in a large sample of Spanish universities over several weeks in 2022, with the participation of hundreds of university students (*n* = 1472). The predictors of suicidal ideation were gender, types of social relationships, history of bullying, health status, taking antidepressant medication, increased anxiety after COVID-19, economic difficulties in continuing studies, and perspective on their future. The results highlight the need for the greater involvement of universities by establishing programs for preventing, detecting, and treating suicidal ideation, always in coordination with health systems to prevent further suicides in their university community.

## 1. Introduction

Death is an event inherent to every living being from birth and is indissoluble to life. What marks the tragic character of this experience is the form and manner of death and the age at which it occurs. If we believe that the death of a baby or a child is unnatural because we understand that a person must develop in life until the body ceases to function, life would be understood more in terms of time than in terms of the experience of life. Suicide, thus, appears as an act in which the time of life itself is shortened [1] for different reasons, such as social, cultural, economic, biological, or psychological.

Suicide is a social and cultural phenomenon [2] that occurs more frequently in Western society. Difficulty seeking help, hopelessness, losses, loneliness, or previous attempts are indicators to consider, because they can trigger suicidal ideation. On the other hand, some factors protect people from this risk: personal relationships, coping resources, prevention, and religious and spiritual beliefs [3].

From a cultural point of view, suicide and the suicide ideation has had several meanings, and in this sense, it has been performed as a form of protest or anger [4,5], as in the case of enslaved people in Mayan society or specific populations in the process of acculturation [6]. The fact that a person puts an end to their life is an act that, if there is no objectifiable element, is not understood and/or accepted by the population. It has been attempted to be deciphered from psychoanalytic, psychiatric, sociological, and even biological perspectives [7,8,9,10,11].

A fact that has been striking recently is the increase in suicide attempts and completed suicide in the university population. The university is an institution of reference for many young people who find a weakening of other structures, such as family, religion, or support networks. In this sense, it is of vital importance to analyze the causes of why young people decide to attempt to end their lives and to what extent the university, as an institution responsible for providing certain services, not only educational, can prevent such behavior and improve resources to prevent the development of suicidal ideas.

## 2. Theoretical Framework

### 2.1. Suicidal Ideation: A Multifactorial Phenomenon

Suicidal behavior is a global problem that is still taboo today. The fear of “social contagion” was made years ago, such that the subject was not talked about in the media, also leaving without visibility the alternatives and tools that could help suicidal ideation not to progress further in the form of attempted or completed suicide.

The WHO defined suicide as any act by which a person causes injury to himself, whatever the degree of lethal intent and knowledge of the real reason for this act [12]. This same organization states that approximately 800,000 people die annually, and many others attempt it [13]. Suicide is among the top three causes of death in people aged 15 to 44 [14].

In Spain, during 2020, suicide remained the leading cause of external death above accidents, drowning, submersion, falls, assaults, and homicides, with a total of 3941 deaths (2930 men and 1011 women), occurring 7.4% more than in 2019. The months of August (34.0% more) and February (28.2%) presented the most significant increases in the number of suicides, according to the Spanish National Institute of Statistics [15]. In the adolescent population in Spain, the prevalence of suicidal ideation is at 30%, and those of suicide attempts are practically 4% [16].

According to several authors [17,18], suicide is a characteristically human and universal fact, the causes of which are due to the presence of several factors, such as genetic, biological, psychological, sociological, environmental, and also cultural. Currently, along with suicide, other physical and mental morbidity factors resulting from failed attempts are considered a public health problem worldwide.

Given that suicidal behavior is multicausal, it is essential to detect risk factors, since the orientation to such behavior can be precipitated by representations of death, thoughts and wishes to die, suicidal ideation, and gestures until reaching the attempt, and often, the completed suicide [19,20,21] and, thus, in this way it can be addressed early to prevent death.

The scientific literature considers several risk factors essential in the approach to suicidal behavior. Suicidal ideation predicts both suicide attempts and suicide. For this reason, it is paramount to keep it in mind, because suicide has it as the main action or starting point [22,23,24,25,26]. Eguiluz [27] defined it as “those intrusive and repetitive thoughts about self-inflicted death, desired ways of dying, and about the objects, circumstances, and conditions in which it is proposed to die”. From a psychological point of view, suicidal ideation is considered a latent variable, i.e., it is not observable to the naked eye and must be inferred [28].

Psychological factors, such as depression, are involved in this stage of suicidal ideation [29,30,31,32,33]. The Spanish Society of Psychiatry notes that the risk of suicide is associated with most mental disorders, especially depression, which is 21 times higher [3]. Between 60% and 90% of people who end up taking their own lives had symptoms of depression. Low self-esteem also plays a role [34], although this statement is controversial, finding publications that show a direct relationship between suicidal ideation and low self-esteem [35] and others that do not [7]. 

Variables to consider in the analysis of this phenomenon are behavioral ones, such as drug and alcohol consumption [14,36] or eating disorders, finding a relationship between risky eating behaviors, low body mass index, and suicidal ideation [32,37].

As for the social factors associated with suicidal ideation, the family context, intrafamily communication, and family support in different circumstances help reduce depression and the risk of suicidal ideation [38,39,40]. 

People who belong to dysfunctional families have a higher suicide rate. Authors such as Ruiz and Orly [41] affirmed that a protective factor against suicide is marriage, because it provides more extraordinary healthy habits and social support [2]. However, this is not always a preventive factor and corresponds more to a cultural element because of what happens worldwide with gender violence or, as Ruiz Pérez and Orly [41] point out, what happens in certain cultures, such as India, Pakistan, or Hong Kong: marriage is a risk factor for female suicide due to the social, legal, and economic discrimination suffered by women in those countries [2].

In addition to the family context, the social framework also plays an essential role in the risk of suicidal behavior. Factors such as psychosocial stress [42], psychological and behavioral control [43], or homophobia [44] help such ideation and the possibility of an attempt to develop. These social risk factors are crucial in the young and adolescent population due to the fact of vulnerability related to pressure from studies, peers, and social networks [45].

### 2.2. Young People and Suicide

The young and adolescent population worldwide presents, in recent decades, an increase in the rates of attempted and completed suicide [46]. In this population, specifically college students, especially between 18 and 24, suicide is the second leading cause of death [14,47,48].

Although there are indeed studies that show that among young people, fatal suicidal behavior is lower than in the population over 65 years of age [49], the figures are still alarming. If we consider that suicidal ideation and suicide are self-inflicted violence, other types of violence, such as school, family [50], or intimate partner violence [51], are for Krug et al. [52] important factors to be careful of as possible risk factors.

As in the adult, adolescent, and young populations, a factor associated with suicidal ideation is related to the social and academic context, since these environments have particular relevance in this crucial period. Accepting peers and friendships and adapting to the environment is necessary for a young person to begin building the pillars of adulthood [53]. This time in their lives is vital for the adolescent and young person to develop the necessary skills and psychological resources both socially and personally to face the day-to-day activities, as they spend many hours of their lives in this context [54,55]. If the environment is not good and relationships are no longer favorable, with psychosocial and academic stress coupled with a hostile family context there is a risk of suicidal ideation and suicide attempts [20,42,56,57].

On the other hand, bullying and intimidation that can be exercised among the peer group within higher educational institutions are significant problems for triggering suicidal ideation. Although cases of bullying in the school environment are more reported, cases in the university community also cause severe damage to the victim, such as posttraumatic stress, low self-esteem, feelings of helplessness and guilt, social isolation, anxiety, and panic attacks [56].

In short, approximately one million people each year commit suicide [58]; at least 20 times that number attempt suicide [59]. Several factors can increase the risk of suicidal ideation in the young population [60,61,62,63], and it is necessary to take them into account to prevent, as far as possible, both the ideation and the attempt and, above all, the consummated act of suicide. 

Several theories can explain this phenomenon, one of which is the three-step theory of suicide by researchers Klonsky and May [64]. This theory identifies the principles of suicide and the conditions under which suicidal thoughts and acts occur. Suicide is explained in terms of four factors: grief, hopelessness, connectedness, and suicidality. Because of the above, it is necessary to speak in terms of prevention, especially in young people, to naturalize mental health intervention. Thus, it is required to present two essential theories in suicide prevention [65]: the interpersonal psychological theory (IPT) of suicidal behavior [66], which postulates that suicidal thoughts arise from high levels of perceived burden and frustrated belonging, and the integrated motivational–volitional model (IMV) [67], which conceptualizes defeat and entrapment as crucial drivers of suicidal ideation.

## 3. Methods

The general objective of this research was twofold: on the one hand, to determine the levels of suicidal ideation in university students and, on the other, whether there are predictors of suicidal ideation in this group.

### 3.1. Study Design

The research was conducted by the University of Murcia and the Complutense University of Madrid between 15 September and 15 October 2022, with the approval of the Ethics Committee of the Complutense University of Madrid (Ref: CE_20220915-09_SAL).

The Plutchik and Van Praag scale was used as a reference in the approach to the object of study [68]. The reason for administering this questionnaire during this period was to isolate intervening stressors linked to the study, given that the students had just started the 2022/2023 academic year in Spain. 

The research design was through simple random sampling since we depended on university managers’ and professors’ involvement in disseminating the survey among the student body. Initially, professors from other universities were contacted and asked to participate in the research. Subsequently, they were informed of the questionnaire and sent the link to access the survey to send it to their students. Moreover, it should be considered that suicide is still a taboo subject in Spain, and the participation of the universities could lead to the presentation of results from their students, an aspect that could be compromising for the university managers themselves. Despite this, the questionnaire was administered onsite and online in more than forty public and private Spanish universities. The national sample and its results can be extended to the whole of Spain, given that universities from all Spanish Autonomous Communities, types of ownership, and teaching methodologies participated.

### 3.2. Variables Used

#### 3.2.1. Dependent Variable

The Plutchik and Van Praag Scale measured suicidal ideation [68]. The Plutchik Scale was included in the questionnaire at the end, after other blocks of questions associated with independent variables, and assesses previous self-harm attempts, the intensity of current suicidal ideation, feelings of depression and hopelessness, and other factors related to suicide attempts.

It is a scale comprising 15 items with 2 response options (yes/no). To establish the existence of suicidal risk, there must be a score of 6 or higher in the yes response option, assigning a value of 1 to affirmative answers and 0 to negative ones. This scale was validated in the Spanish population [69], with a Cronbach’s alpha of 0.89 and sensitivity and specificity of 88%, to differentiate between people with and without a history of suicide attempts.

#### 3.2.2. Independent Variables

In our study, we used other types of variables: (a) sociodemographic variables, (b) family and coexistence variables, (c) quality of social relations, (d) subjective assessment of health, and (e) economic capacity to meet the expenses derived from university studies.

### 3.3. Participants

The sample reached 1472 students with a mean age of 24.1 years and a median of 21.0 years. By gender, 84.6% were women, 14.3% were men, and 1.1% were nonbinary. Regarding the sample by universities, the universities with the highest level of participation were the University of Murcia (26.1%), Universidad Nacional de Educación a Distancia (15.9%), Universidad Complutense de Madrid (13.8%), Universidad de País Vasco (5.2%), Universitat de Valencia (4.9%), Universidad de Castilla la Mancha (4.7%), Universitat de Les Illes Balears (3.4%), Universidad de Granada (2.9%), and Universidad de Deusto (2.8%).

### 3.4. Procedure

First, a descriptive analysis of the most representative variables was carried out based on sociodemographic aspects, household composition, and parents’ level of education. Subsequently, a study was made of the responses to the Plutchik scale to observe the most relevant items. 

Finally, to determine the predictive variables of suicidal ideation in university students, a binary logistic regression was performed using the intro method, taking suicidal ideation as the reference and dependent variable. The computer program used to develop this analysis was IBM SPSS v. 24. The independent variables introduced in the binary logistic regression are shown in Appendix A.

## 4. Results

The descriptive analysis shows the following results (Table 1). Firstly, the sociodemographic variables showed a feminization of the participants, with 84.6% being women. In terms of gender, 1.1% considered themselves to be nonbinary. If we distribute by age, most of the participants were up to 25 years old (80.3%). For this reason, an age distribution was conducted for those up to 19 years of age, who accounted for 28.3%; 19–22 years of age, which reached 39.6%; and 23–25 years of age, whose records were reduced to 12.4%. Thus, it can be seen that the population participating in this study was very young, with 67.9% of those up to 22 years of age.

Regarding marital status, 81.1% were single, and 95.8% had Spanish nationality. Most of the students lived in their family home of origin (69.5%) and, secondly, in shared apartments (19.5%). Of these, 49.3% did not perform any work activity.

Of the parents, 67.0% were married, and 23.2% were separated or divorced. In the case of the parents’ highest level of education, an almost proportional distribution was observed in the case of the mother, where 36.8% had primary education (up to the critical stage), 31.9% had secondary education (baccalaureate, intermediate vocational training, etc.), and 31.3% had higher education (university or higher vocational training). On the other hand, concerning men, the percentage of those with primary education increased compared to women, reaching 41.3%, and those with higher education (24.6%) decreased, while the level of secondary education remained at the same levels (34.1%).

Subsequently, we delved into the variables that may have more excellent explanatory value as independent variables, such as those related to the quality of social relations and subjective assessment of health, as well as the economic capacity to meet the expenses derived from university studies. Regarding the types of social relations, it is noteworthy that socialization in the classroom in onsite universities—online universities were not considered due to the distorting effect of the comparison—did not represent a protective element; however, on the contrary, social relations worsened inside the classroom. Thus, of the participants who stated that they had satisfactory social ties (in general), 80.0% reduced this evaluation to 59.7% when they confined themselves to the academic environment. A total of 5.7% stated that they had no relationships in the classroom, and 34.6% considered them indifferent.

On the other hand, a series of results are presented below that, in themselves or jointly, can act as stressors for university students’ mental health. On the one hand, 34.0% stated that they have suffered bullying in previous stages, a very significant figure, and 7.7% take antidepressant medication; of these, 1% had been on antidepressant medication for less than 2 months, and 6.7% had been on medication for more than 2 months. Some 53.3% had an increased level of anxiety concerning their studies after COVID-19, and 54.1% received information concerning suicide. In addition, 31.0% had financial difficulties in continuing their university studies. 

Next, we related the subjective assessment of the current state of health and their projects. First, it should be noted that most students were in good health, and their prospects were also positive. However, those who rated their health situation as very bad (0.9%) or bad (2.4%) practically doubled their scores when asked about their prospects, rising to 1.6% and 5.6%, respectively. These data are significant because the future outlook is framed with their activity in the university where they are training to develop a professional activity. As in previous cases, poor health and a poor assessment of their future impact university studies and the student’s mental health.

The Plutchik scale presents a series of items with the same value when calculating suicidal risk, but it is convenient to highlight which are more representative, as shown in Appendix B. When applying the scale, we obtained a very high value for suicidal ideation since 32.4% obtained positive values.

Of all the values on the scale, those with the highest positive percentages were (a) have ever felt useless or useless (75.2%), (b) have ever felt such a failure that they just wanted to get into bed and give up everything (67.0%), (c) have difficulty falling asleep (45.4%), (d) sometimes feel that they could lose control over themselves (40.1%), and (e) have ever thought of committing suicide (36.7%). In addition, of these very high values, it is noteworthy that 9.7% of the students had attempted suicide at some time; that is, 1 out of every 10 university students has had a self-injury attempt.

Following the methodology described above, we used the binary logistic regression technique to evaluate whether there are predictive factors in suicidal ideation by university students. The dependent variable was suicidal ideation (yes/no) in university students. The set of independent variables used in the binary logistic regression is shown in Table 1.

The logistic regression model was statistically significant: X^2^ = 520.393, *p* < 0.001. The model explained 44.3% (Nagelkerke’s R^2^) of the variance in the dependent variable of risk of suicidal ideation and correctly classified 80.3% of the cases. The Hosmer–Lemeshow test showed no significant difference between the observed and predicted results in the model, with a *p* = 0.646. 

Based on these results, the minor significant variables were eliminated using the automatic “Wald: stepwise advance” method. From the set of independent variables introduced in the binary logistic regression, those that showed predictive capacity were the following: (a) gender (nonbinary), (b) social relationships (indifferent/nonexistent), (c) bullying in previous stages of schooling (yes), (d) health status (fair/bad/very bad), (e) taking antidepressant medication (for more than 2 months), (f) has increased her level of anxiety in her studies after COVID-19 (yes), (g) has economic difficulties to continue her studies (yes), and (h) perspective on her future (good/regular/bad) (Table 2).

In the specific case of gender, people who defined themselves as “nonbinary” presented an OR = 12.254, IC 95% (2.121–70.795), *p* = 0.005. Therefore, the risk of suicidal ideation was 12 times higher in men. Furthermore, taking gender into account, it should be noted that no significant differences were obtained in the case of women compared to men, so the “nonbinary” category was the only one representative of gender.

With the type of social relationships (general and academic), the data showed an OR = 2.062, 95% CI (1.402–3.032), *p* < 0.001. Therefore, those whose social relationships were characterized by “nonexistence/indifference” risk suffering suicidal ideation twice as high as those with satisfactory relationships.

Bullying, one of the main scourges of society and the educational system [70], is also a predictive variable of suicidal ideation. Those who report having suffered bullying in stages before university showed an OR = 2.602, IC 95% (1.942–3.488), *p* < 0.001; that is, they had 2.6 times greater risk of suffering suicidal ideation than those who had not suffered it.

Health status was another of the predictive variables that showed a greater risk of suffering suicidal ideation in three of its categories: “fair”, “bad”, and “very bad”. Concerning the subjective assessment of “fair” health, it presents an OR = 22.867, IC 95% (6.845–76.397), *p* < 0.001. In the case of the subjective assessment of “bad”, it presented an OR = 6.918, IC 95% (3.622–13.213), *p* < 0.001. Finally, within this variable, the subjective evaluation of “very bad” showed an OR = 2.513, IC 95% (1.363–4.633), *p* = 0.003. Therefore, those who considered their state of health to be “fair” were 22.8 times more likely to suffer suicidal ideation than those who thought their state of health to be very good; 6.7 times more likely in the case of people who rated their health as “bad”; and 2.5 times more likely in the case of people who rated their health as “very bad”. These data indicate that the fact that university students did not rate their health significantly negatively does not mean that it was not indicative of suicidal risk, i.e., the simple point of not considering their health “good” or “very good” was a predictive factor. In other words, students did not have to be in very poor health to be at increased suicidal risk, as the “fair” health status data show.

In the case of “if taking antidepressant medication: for more than 2 months”, it presented an OR = 6.418, IC 95% (3.329–12.372), *p* < 0.001. Therefore, people who maintained over time an antidepressant medication had a risk of suicidal ideation up to 6.4 times higher than people who “do not take antidepressant medication”. At this point, it should also be noted that taking antidepressant medication for less than 2 months did not appear as a predictor variable, an aspect that indicates the relevance of the chronicity of antidepressant treatment in suicidal risk.

COVID-19 generated increased stress and anxiety for the population, and this study indicates that it also occurred in university students, acting as a predictor variable for the risk of suicidal ideation. Those who had seen “increased anxiety after COVID-19” presented an OR = 2.102, IC 95% (1.553–2.844), *p* < 0.001; that is, these university students had a two times greater risk of suffering suicidal risk than those who did not experience an increase in their level of anxiety as a result of the COVID-19 pandemic.

In Spain, university education is not free, although scholarships are available to university students. However, there are considerable expenses associated with studying that are not covered. Economic difficulties also emerged as a predictive variable when it came to being able to cover the costs derived from university studies. This variable presented an OR = 1.927, IC 95% (1.428–2.600), *p* < 0.001. Therefore, these students, who may have had more economic difficulties, saw their risk of suicidal ideation increase by almost two times; thus, financial capacity or economic lack acted as an explicit stressor.

The last predictor variable was the outlook for the future, in the categories “good”, “fair”, and “bad”, concerning people who had a “very good” evaluation. In the case where perspective on the future was “good”, it presented an OR = 7.530, 95% CI (2.082–27.232), *p* = 0.002. Regarding those whose perspective on the future was “fair”, they provided an OR = 7.956, IC 95% (3.665–17.271), *p* < 0.001. Finally, those who had a “poor” assessment of their future had an OR = 2.850, IC 95% (1.670–4.863), *p* < 0.001. These data indicate that the risk of suicidal ideation was high in cases of “good” and “fair” perceptions of the future, with a 7.5 and 7.9 times greater possibility of suffering this phenomenon, respectively. Thus, a positive view of the future will not act as a compensating factor in situations of suicidal ideation but will be present, although it may not necessarily be a determining factor. In this sense, expectations about the future or those not fulfilled during the educational stage due to the fact of unavoidable circumstances may come into play, including failure of subjects, illness, and lack of economic capacity. All these circumstances can act in the opposite direction, favoring frustration and are stressors to be considered.

## 5. Discussion

Our study highlights that 9.7% of students who responded reported having attempted suicide. That is, 1 out of every 10 university students has had a self-inflicted suicide attempt. The research before COVID-19 showed significantly lower figures proportionally to the data obtained in our study. Blasco et al. [71], from a sample of 2118 first-year students from all subject areas, showed a 12 month prevalence of suicidal thoughts and behavior of 9.9; 5.6 had a suicidal plan, and 0.6 attempted suicide. In our study after the binary logistic regression, the variables that showed predictive ability were the following: (a) gender (nonbinary), (b) social relationships (indifferent/nonexistent), (c) bullying in previous stages of schooling (yes), (d) health status (fair/bad/very bad), (e) taking antidepressant medication (for more than 2 months), (f) has increased their level of anxiety in studies after COVID-19 (yes), (g) has economic difficulties to continue their studies (yes), and (h) perspective on their future (good/regular/bad). In this case, the innovative results are presented based on other research results.

For example, Miranda-Mendizabal et al. [34] pointed out that the main factors favoring suicidal ideation were mood disorder throughout life, which we could say is in line with a factor detected in our study (i.e., taking antidepressant medication for more than 2 months). From a gender perspective, in females, they included exposure to parental violence, anxiety disorder, and alcohol/substances. In the case of males, the predictive factors of suicidal ideation that the study showed were physical abuse/child abuse, having deceased parents, and hopelessness. Family, family support, and peer/other support were associated with a lower risk of suicidal ideation only among females. Addressing the issue of bullying and its direct relationship with low self-esteem, some studies show that in the university population, there is less suicidal risk the higher the intelligence, clarity and emotional regulation, self-esteem, and self-confidence they have [46,72,73,74] and that low self-esteem increases by up to three times the risk of suicidal ideation and attempts [75].

In one study [76] with a multiple logistic regression analysis in a university in Bangladesh, among the variables that were established, being female, being a fifth-year student, a lower socioeconomic level, exposure to traumatic events, and family history of suicide and depression were associated with suicidal ideation, highlighting the sociopolitical and cultural context of the country. The cultural context and the low-income [77] socioeconomic reality were determining factors in our research and in others conducted worldwide. In this sense, the socioeconomic situation of indigenous students in the United States or Canada determines the high rates of suicidal ideation and suicides in this minority population [78]. A study conducted in 12 countries [79], with a sample of 5572 university students, showed that suicidal ideation, suicide attempts, and psychological distress are common in university students, but their rates vary according to the sociocultural context. The odds of suicidal ideation were high in Austria and the United Kingdom and low in China, Italy, Saudi Arabia, Tunisia, and Turkey. Similarly, while the odds of attempted suicide were high in Jordan, Palestine, Saudi Arabia, and, to some extent, Turkey, they were low in Austria, China, Italy, Japan, and the United States [80].

The previous studies analyzed [77,78,79] focused on first-year students given the stress experienced by young people starting university, but in our sample no differences were found between ages, i.e., between those entering university for the first time and those who have been there for more years. This indicates that the difficulties of adaptation and prevalence of suicidal ideation have to be treated during the entire teaching–learning process experienced by students at the university, considering the presence of the predictive factors mentioned above.

The existence of positive relationships in the university classroom among classmates and teachers and the support and good communication in the family are considered protective factors against STS [80,81]. This assessment contrasts with the fact that students’ relationships in the classroom are worse than those in a general context. Thus, the responses of nonexistent or unsatisfactory social relationships in the classroom increased. According to the results of our study, 5.7% said they have no relationships in the classroom, and 34.6% considered them indifferent. In a study of Chinese university students aged 17 to 24, three elements related to unsatisfactory relationships in the classroom (frustrated belonging, perceived burden, and acquired capacity for self-harm) were established [82]. 

The students rated their health situation as very bad (0.9%) or bad (2.4%), practically doubling their scores when asked about their prospects, rising to 1.6% and 5.6%, respectively. These data are significant because the future outlook is framed with their activity in the university where they are training to develop a professional activity. As in the previous cases, poor health and poor assessment of their future impacted their university studies and mental health.

The variables with the most significant predictive capacity were nonbinary gender (up to 12 times more). This variable was studied in a sample of 2778 students belonging to Canadian sexual and gender minorities [83,84], highlighting the emotional fragility of people in this situation [85]. In research on UK university students (with a sample of *n* = 707), an LGTBQ status remained associated with an elevated risk of nonsuicidal self-harm and suicide attempts [86]. These results are connected to recent research by Call and Shafer [87] that showed that LGTBQ people are at risk for suicidal behaviors. 

The proposal to incorporate the “nonbinary” variable is novel and sheds light on the fact that being a woman has always meant a greater disposition to suicidal ideation. Recent studies have shown the differences in suicidal behavior between men and women. Men have more completed suicides than women (3:1 ratio), while suicide attempts place women at a 3:1 ratio concerning men. In samples of adolescents and young adults, females have a higher risk of attempted suicide than males of completed suicide. In addition, females commit twice as many suicide attempts as males, and suicide is the leading cause of mortality in young girls aged 15–19 years globally [88]. Except in China, in most countries, the suicide rate in men is 2–4 times higher than in women [88], suggesting that many men have undiagnosed mental health problems [89]. However, no such differences between men and women were evident in our study.

On the subjective assessment of fair or poor health status (22 and 6.9 times more) and taking antidepressant medication chronically (6.4 times more), it should be linked to the deficits existing in Spain in mental health care [90,91]. Although the future perspective also collects relevant data, the disparity and diversity of its response categories mean that, generally, it cannot be suitable, as shown in Section 4. Taylor points out the importance of early intervention, focusing on becoming less critical or self-critical of oneself and, thus, preventing this negative future perception as a possible contributing factor to suicidal ideation [86]. This reality, in some cases, is linked to the perception they have of their body, this not being a determining factor but their state of depression [92]. In this regard, the White Paper on Depression and Suicide [3] in Spain points out that “the basis of effective prevention is the approach to risk factors, identifying and mitigating them, the use and enhancement of protective factors and the improvement of the health system”.

In addition, 7.7% of university students took antidepressant medication; of these, 1% had taken medicines for less than 2 months and 6.7% for more than 2 months. A total of 53.3% had an increased level of anxiety concerning their studies after COVID-19, and 54.1% had received information about suicide. People with moderate/high anxiety and depression were linked to a risk of suicidal behaviors. In addition, 31.0% had financial difficulties in continuing their university studies [93].

## 6. Conclusions

Society maintains a series of discomforts that generate social discontent, where people who suffer may resort to suicidal ideation to stop suffering. The measures developed by the university in favor of its students must be inserted and coordinated with the national health system and/or mental health centers. The lack of coordination or appropriate resources generates social and personal risk for those with social or psychological difficulties in coping with their daily lives, making it difficult to curb the number experiencing suicidal ideation in this group. 

The figures obtained in our study maintain the results of previous studies on university students, and society cannot remain paralyzed in the face of these elevated results of suicidal ideation. Most research findings on college students emphasize the need for appropriate support services for college students, with a focus on mental health wellness and suicide prevention [59,85].

Spanish universities should look at the realities in other countries and implement their prevention and treatment mechanisms, as European and American universities have. Attention should be paid to the mental health needs of young adults in higher education institutions, and more cross-cultural research should be conducted to understand better the etiology of the intersocial variations observed in suicidal behavior and psychological distress [94]. Thus, as an institution that pursues knowledge, research, and transfer to society, the university should not be oblivious to this reality and should activate health and wellness care devices for students while designing action plans coordinated with existing mental health devices. 

Enacting a suicide prevention law with a budget for creating services with qualified professionals would help reduce the current situation [65]. These services should be coordinated with existing care, counseling, or training services in schools and universities. 

In addition to the above, systematic violence in the educational system should be stopped by establishing measures to eliminate bullying or school harassment and, thus, avoid one of the causes that can encourage suicidal ideation, according to the results obtained in our research. At the same time, teaching and learning methodologies are designed to favor good relationships in the classroom, given their value as a restorative effect or containment of suicidal behavior. The feeling of frustrated belonging in the classroom can be a determining factor that university teachers can modify to contribute to reducing the perception of dissatisfaction or maladjustment in the university and to be a factor in the containment of suicidal ideation. 

In general, the development of this work has allowed for a comprehensive and circular presentation of a problem with high health, social, and educational repercussions: suicidal ideation and suicide within a social and cultural framework; suicidal ideation from a clinical point of view; youth, university, and suicidal risk; suicidal risk and predictive factors; and the role that universities should play in coordination with health systems according to the results obtained. 

## 7. Limitations

In general, self-reports are subject to prejudices and limitations. It may be that the subject leads to socially acceptable answers beyond a respondent’s sincerity or that they lack an introspective ability and may not be able to accurately evaluate themselves. However, in our study and due to its nature, these drawbacks were minimized so that their incidence, in any case, would be unimportant. 

On the other hand, and although only a sample of universities was studied, their geographic dispersion in Spain was established. The characteristics of the sample of participating universities were very similar to those of Spanish university students. In the study, the participants responded without receiving monetary incentives so as not to introduce bias in the information obtained. 

## Figures and Tables

**Table 1:** Descriptive analysis of sociodemographic, family, and cohabitation variables.

	Frequency	%
Sex		
Woman	1246	84.6
Male		14.3
Nonbinary		1.1
Age		
Up to 19	416	28.3
20–22	583	39.6
23–25	182	12.4
26–30		5.7
31–40		7.1
41–50		5.2
51 and over		1.7
Marital Status		
Single	1194	81.1
Unmarried partner		10.7
Married	99	6.7
Other		1.1
Nationality		
Spanish	1398	95.8
Foreign		4.2
Coexistence		
Family home	1021	69.5
University residence		1.7
Shared apartment	287	19.5
Only		4.6
Another		4.8
Currently working		
No	724	49.3
Yes, sporadically (<3 months per year)	231	15.7
Yes, sporadically (>3 months per year)		9.5
Yes, permanently		25.5
Marital Status Parents		
Married/living with a partner	985	67.0
Separated/divorced	341	23.2
Widower		7.7
Other		2.1
Studies Mother		
Primary	539	36.8
Secondary	468	31.9
Superiors	459	31.3
Studies Father		
Primary	596	41.3
Secondary	493	34.1
Superiors	355	24.6

**Table 2:** Logistic regression results.

	B	Sig.	Exp(B)	95% CI for Exp(B)	
				Inferior	Superior
Gender: nonbinary	2.506	0.005	12.254	2.121	70.795
Social relationships: indifferent/nonexistent	0.724	<0.001	2.062	1.402	3.032
Bullying previous stages of schooling: yes	0.956	<0.001	2.602	1.942	3.488
Health status: fair	3.130	<0.001	22.867	6.845	76.397
Health condition: poor	1.934	<0.001	6.918	3.622	13.213
Health status: very poor	0.921	0.03	2.513	1.363	4.633
Taking antidepressant medication: for more than 2 months	1.859	<0.001	6.418	3.329	12.372
Has your level of anxiety in your studies increased after COVID-19: yes	0.753	<0.001	2.102	1.553	2.844
Do you have financial difficulties continuing your studies: yes	0.656	<0.001	1.927	1.428	2.600
Outlook for the future: good	2.019	0.002	7.530	2.082	27.232
Outlook for the future: fair	2.074	<0.01	7.956	3.665	17.271
Outlook for the future: poor	1.047	<0.01	2.850	1.670	4.863
Constant	−4.125	<0.01	0.016		

## Data Availability

Data are available upon request due to the fact of privacy and ethical restrictions. The primary data are contained within this article.

## Appendix A. Independent Variables Introduced in the Binary Logistic Regression

1. Gender	9. Maximum mother’s studies
Ref. Male	Ref. Superior
(1) Female	(1) Media
(2) Nonbinary	(2) Mandatory
2. Age	10. Assessment of social relations
Ref. 51 and more	Satisfactory Ref.
(1) 41–50	(1) Indifferent or nonexistent
(2) 31–40	11. Classroom environment
(3) 26–30	Ref. Satisfactory
(4) 23–25	(1) Indifferent or nonexistent
(5) 20–22	12. Bullying in previous educational stages
(Up to 19)	Ref. No
3. Marital Status	(1) Yes
Ref. Separated/divorced	13. Assessment of your health status
(1) Domestic partnership	Ref. Very good
(2) Single	(1) Good
(3) Married	(2) Regular
4. Nationality	(3) Bad
Ref. Spanish	(4) Very bad
(1) Foreign	14. Takes antidepressant medication
5. Currently working	Ref. No
Ref. Yes, permanently	(1) Yes, less than 2 months ago
(1) Yes, temporarily (more than 3 months per year)	(2) Yes, more than 2 months ago
(2) Yes, sporadically (less than 3 months per year)	15. You have received information about suicide
(3) No	Ref. No
6. Marital status of parents	(1) Yes
Ref. Married/living with a partner	16. Your anxiety level has increased as a result of COVID-19.
(1) Separated/divorced	Ref. No
(2) Widowed	(1) Yes
(3) Other	17. You have financial difficulties paying for university studies
7. Coexistence	Ref. No
Ref. Family home	(1) Yes
(1) University residence	18. Perspective on your future
(2) Shared apartment	Ref. Very good
(3) Only	(1) Good
(4) Other	(2) Regular
8. Maximum mother’s studies	(3) Bad
Ref. Superior	(4) Very bad
(1) Media	
(2) Mandatory	

## Appendix B. Values of the Plutchik Scale Items

	n	%
Do you regularly take any medications, such as aspirin or sleeping pills?		
No	1293	87.8
Yes		12.2
Do you have difficulty falling asleep?		
No	804	54.6
Yes	668	45.4
Do you sometimes feel that you might lose control over yourself?		
No	881	59.9
Yes	591	40.1
Do you have little interest in relating to people?		
No	1070	72.7
Yes	402	23.7
Do you view your future with more pessimism than optimism?		
No	1030	70.0
Yes	442	30.0
Have you ever felt useless or worthless?		
No	365	24.8
Yes	1107	75.2
Do you see your future as hopeless?		
No	1342	91.2
Yes		8.8
Have you ever felt like such a failure that you just wanted to crawl into bed and abandon everything?		
No	486	33.0
Yes	986	67.0
Are you depressed now?		
No	1204	81.8
Yes	268	18.2
Are you separated, divorced, or widowed?		
No	1430	97.1
Yes	42	2.9
Do you know if anyone in your family has ever attempted suicide?		
No	1041	70.7
Yes	431	29.3
Have you ever felt so angry that you would have been able to kill someone?		
No	1286	87.4
Yes	186	12.6
Have you ever thought of committing suicide?		
No	932	63.3
Yes		36.7
Have you ever told anyone that you wanted to commit suicide?		
No	1148	78.0
Yes	324	22.0
Have you ever tried to take your own life?		
No	1329	90.3
Yes		9.7
The suicidal Ideation Scale score		
Yes	477	32.4
No	995	67.6
